# Letter from the Senior Editor

**Published:** 1845-08

**Authors:** 


### THE WESTERN JOURNAL.

NEW SERIES.—VOL. IV.—NO. II.

LOUISVILLE, AUGUST 1, 1845.

### FROM THE SENIOR EDITOR.

CINCINNATI, June 23d, 1845.

*To my Colleagues:*

GENTLEMEN—

Having waited until our readers may have so far recovered from my epistolary doses of last summer and fall, as to bear a renewal of them, I have concluded to re-commence their administration with the opening of our 10th volume. To prevent alarm, however, both on your part and theirs, I will state that I expect to deal, altogether, in homœopathic portions, at least as far as any thing requiring much mental digestion is concerned. The temptations to this are always strong to one who has but little to dispense, but in this city of homœopathism the brilliant success of that system invites to its universal adoption. If a man be ambitious of praise, (not the applause of the vulgar, who guzzle down the newest patent purging pills, or the latest decoction of Dacotah *makha*, modified with the essence of bear's oil,) I say, if any one aspires after the admiration of first-rate ladies and gentlemen, and his lot be cast here, let him turn homœopathist; if he would drive through the streets in a barouche, while the "reg'lar" doctor trundles in a buggy, let him label it with that cabalistic term; if he would live in a splendid mansion, and point to it as a monument of his professional populartity, let him write the magical word underneath his name, when he is about to hang out his "shingle." Homœopathy is, *par excellence*, a quackery for the higher classes—the intellectual and refined, whose stomachs recoil from bulky and bitter draughts, while their imaginations supply the place of active medicinal qualities, when they are laboring under two groups of maladies, the incurable and the insignificant. Homœopathy is scientific empiricism; and its popularity with those who would reject every kind of coarse and vulgar quackery, has answered, in the *negative*, this momentous question—can wealth and refinement raise a people above the region of credulity? The high, not less than the low, require to be imposed upon, but the imposture must be adapted to their refined taste. If this be true of the *elite* of this city, it is equally so of those of St. Louis and Louisville. It is the infirmity of human nature, and not of the Queen city only.

You must not suppose from what I have said, that the good people here swallow quackery in homœopathic doses only. So far from it are they, that the city abounds in warehouses, almost rivaling those for pork, in which are treasured up exhaustless supplies of decoctions, balsams, elixirs, lozenges, tinctures, honies, plasters, and linctuses, sending forth odors and stenches sufficient to advertise the way-faring man of their contiguity in the darkest night; when the vast handbills which adorn our walls cannot be read. Other modes of advertising besides these and the daily papers, (in which things unutterable are blazoned forth), are red and yellow pamphlets, which their authors distribute in the form of tracts. Two of these, just handed in, are now before me; and according to their own modest testimony, render it unnecessary for me to proceed further, in the labor of collating my notes of the experience of our physicians on the diseases of our great valley. Both of these interesting little books, I find, are of New York origin. One says on its outer title—"Keep this for future reference;" the other, with greater shrewdness, has inserted an almanac for 1845, in the midst of its thrilling narratives of cures, and its veracious certificates, from an ex-governor of the Empire State, up to his "Highness the Sultan of Muscat." I observe, however, that the 12 signs of the zodiac with their benign and malign influences over the human body, and the daily predictions of the weather are omitted. In the next edition, it is to be hoped they will be reinstated, when the work will be a complete manual of health.

In the war of rival quackeries, I am sorry to find that therapeutic mesmerism has been "knocked down and dragged out." Whether it is dead or only in a state of somnambulism, *I* cannot say, and *nobody* seems to care. In fact, it appears to be forgotten. This is a great pity, and I hope some one of our mesmerizing readers, will be able, by the force of his will, to rouse it into life and action, that it may again take its chance among the grosser charlatanries of the day. If not the least absurd, it is certainly the most harmless of the whole—the most spiritual—and the most auspicious to the tender relations, as it requires the doctor and the patient to be of opposite sexes. One might have thought that this requirement alone would have made it fashionable and preserved its life.

Among the latest inscriptions which I have read on the brazen face of the harlot Charlatana, is the following, which I transcsibe for the benefit of the afflicted. "Office for Diseased Lungs, Bronchitis and Asthma. Open daily, from 8 to 2. Closed on Sunday." It hangs at the corner of Vine and Baker streets. Of its author I know nothing, except that he is said to belong to the scientific fraternity. That he will be able to assemble around him a host of incurables cannot be doubted; and I have as little doubt, that he will soon have rivals here and elsewhere. Indeed, it seems remarkable, that many doctors have not had the stethoscope painted on their signs long ago. A red cedar cylinder is certainly more picturesque than a cast iron mortar and pestle! But I must ask your attention to matters of a different kind.

### *Ohio College of Dental Surgery.*

An institution under this title was incorporated by the Legislature of Ohio, during its last session, with a Board of nine Trustees, of whom about one-half are physicians. Its Faculty consists of the following practical dentists, all advantageously known here for their skill and science.

JESSE W. COOK, M.D., D.D.S.,
*Professor of Dental Anatomy and Physiology, and Dean.*

MELANCTHON ROGERS, M.D., D.D.S.,
*Professor of Dental Pathology and Therapeutics.*

DR. JAMES TAYLOR, D.D.S.,

*Professor of Practical Dentistry and Pharmacy.*

To which may be added that of J. P. Judkins, M.D., as Demonstrator of Anaıomy and Lecturer on Descriptive and Surgical Anatomy.

It is contemplated to add a chair of Chemistry. The first session, to continue four months, will commence on the first Monday of November next. The terms of admission are:—

The matriculation ticket $5. The ticket of each Professor for the session $25. Dissecting ticket (optional) $10.

Diploma fee $25. The fees for a full course $100 (exclusive of the diploma fee), to be paid in advance.

Desirous of making known this laudable enterprise, I transcribe the following terms of graduation:

"Candidates for graduation will be required to have attended two full courses of lectures, the last of which shall have been in this institution.

"A full course of lectures in the Baltimore College of Dental Surgery, or a full course in a regular medical college, will be acknowledged as an equivalent to a course in this institution. The candidate must be twenty-one years of age, of good moral character, and have studied the profession two years with a reputable practitioner in dentistry.

"A regular student of medicine, who has studied one year or more, and has taken a full course of lectures in a regular medical college, may be a candidate, after studying dentistry one year with a reputable practitioner, and taking one full course of lectures in this institution.

"A reputable practitioner in dentistry, who has been four years or more in practice, shall be entitled to an examination for a degree after attending one full course in this college.

"Each candidate will be required to present and defend before the faculty, a written thesis on some subject relating to dental science, and be subject to a critical examination upon the theory and practice of dentistry."

It appears from other parts of the circular, that anatomy and physiology, dental pathology and therapeutics, pharmacy and practical dentistry, will be taught. This, I believe, is the second (that of Baltimore being the first) College of Dentistry established in the United States. I cannot but wish the gentlemen who have projected it, the greatest success, not on their own account so much as that of the community; and that they will, by their talents and exertions, merit the patronage of all who aim at dental surgery as a profession, I have no doubt.

### *Medical Club.*

It would fill out my sheet, to write an epitaph on each of the Medical Societies, which have been 'blighted in the bud,' and withered without bringing forth fruit, in this metropolis within the last thirty years. At present, there is, I believe, but one, and it embraces less than half the physicians of the city. The qualification for admission (after regular graduation and the establishment of a sound moral character) appears to be a capacity for eating a good supper, with an aromatic cup of coffee, at 10 o'clock; beyond which the club does not continue its intellectual labors. The event which gave it birth was, I think, the meeting, here, of the Medical Convention of Ohio, in strawberry time, 1842. It meets monthly, at the houses of the different members, and he who is thus honored, provides the entertainment. One or two short papers are read at each meeting, and their discussion is made a sort of preliminary exercise, to the discussion of the viands prepared by 'mine host.' I lately had the honor of an invitation to one of the meetings, at the house of a medical friend. I did not get there in time to hear the paper on Cancer, (a good one I was told,) by professor Worcester; but when I entered, the first thing I heard, was the sonorous and earnest voice of our learned friend, Professor Harrison, as in times gone by depicting the gross humors and peccant despumations of the humoral pathology, a few drops of which I judged had found its way into the paper. I am told that the Professor, who has the distinction of having written the first work on materia medica in the valley of the Mississippi, has borne your review of it with magnanimity; but is a little disappointed that you had not the courage to 'break a lance' with him on the great question of exclusive solidism. We shall expect, when his second volume, now through the press, makes its appearance, to see a small *bellum pathologicum*—at least, I don't see exactly, how you can back out.

### *Medical Convention of Ohio.*

The annual meeting of the physicians of Ohio, was held in Columbus on the 20th, 21st, and 22d of last month. I had not the advantage of attending, but learn through the newspapers (which do not tell us the names of the officers) that 88 physicians from 30 counties (one third of the State) were present. Professor St. John delivered a public discourse on 'A knowledge of the difficulties which beset the practice of medicine, as essential to the guardianship of the public health;" and Prof. Mussey another on the "Homœopathic practice of Medicine." A specimen of valvular disorganization of the heart, from a boy 10 years old, who died suddenly was exhibited; and leave asked, but not granted, to introduce a woman with some disease of the eye, who was anxious to submit her case to a vote of the convention. Another and perhaps still more difficult case, was, however, discussed at length:—whether the assistant physician of the lunatic asylum ought to be permitted to marry? I am sorry to say. that this appeal of a solitary, to the sympathies of his brethren, was at last 'ruled out of court,' and that he did not obtain permission to go a courting. The members, in a body, visited the asylum, the penitentiary, and the schools for the deaf and blind, and found the whole, as they really are, admirable. An unsuccessful attempt was made to get up a memorial to the Legislature for a law to regulate the practice of physic; and another to organize a State Medical Society, instead of popular conventions, experienced the same fate; as did a resolution on the subject of vending quack medicines;—three judicious decisions. Some of the members furnished lists of the regular and irregular members of the medical profession in their respective counties, to show the progress of empiricism.

Of matters strictly scientific, I may mention the appointment of a committee to make a report, at the next meeting, on the localities, cause and treatment of *milk sickness;* that our friend, Dr. Dawson, was requested to furnish for publication a paper on epidemic erysipelas, concerning which several members made oral statements; and finally, that Professor Mussey addressed the convention on the comparative influence of animal and vegetable food, insisting that man is naturally herbivorous, and that the use of meats is a corruption of his tastes and habits. His address was followed by a discussion, at the close of which the convention ordered his paper to be published.

During the session, the members partook of a supper, at the 'Neil house,' provided by the Medical and Surgical Association of Columbus. Having finished its business, it adjourned to meet at the same place, on the last Tuesday in May, 1846.

### *Summer Lectures.*

Courses of summer lectures, by several of the younger physicians of this city, were commenced, in the month of April, and abandoned in a few weeks. Of the difficulties which beset the enterprise, I am not sufficiently informed to say anything. There are no lectures at Lexington this summer, I believe; so yours are all, I presume, that the Valley of the Mississippi can boast. It is lamentable, that so few of our students are inclined to avail themselves of summer lectures, when the length of time and the small number of lectures daily, would afford them much time for reading and reflection, in connexion with oral instruction.

### *Gross's Pathological Anatomy.*

I have just received a letter from our friend, Professor Gross, whose Elements of Pathological Anatomy in a new and greatly enlarged edition is passing rapidly through the press. This edition will have all the advantages of a fine mechanical execution. It will be in market about the first of September, when I hope you will make known to our readers its various merits and demerits.

I must now close. I fear you will think I am still a polypharmist rather than a homœopathist. Which ever you may regard me,

I remain your very ob't. serv't.,

